# Single-scan volumetric imaging throughout thick tissue specimens by one-touch installable light-needle creating device

**DOI:** 10.1038/s41598-022-14647-3

**Published:** 2022-06-21

**Authors:** Ching-Pu Chang, Kohei Otomo, Yuichi Kozawa, Hirokazu Ishii, Miwako Yamasaki, Masahiko Watanabe, Shunichi Sato, Ryosuke Enoki, Tomomi Nemoto

**Affiliations:** 1 Faculty of Medicine, Hokkaido University, Sapporo, Japan; 2 Exploratory Research Center on Life and Living Systems (ExCELLS), National Institutes of Natural Sciences, Okazaki, Japan; 3 National Institute for Physiological Sciences, National Institutes of Natural Sciences, Okazaki, Japan; 4 Graduate School of Medicine, Juntendo University, Tokyo, Japan; 5 Institute of Multidisciplinary Research for Advanced Materials, Tohoku University, Sendai, Japan; 6 School of Life Sciences, Graduate School of Advanced Studies (SOKENDAI), Okazaki, Japan

**Keywords:** Fluorescence imaging, Ca2+ imaging, Multiphoton microscopy, Biophotonics, Neural circuits

## Abstract

Biological tissues and their networks frequently change dynamically across large volumes. Understanding network operations requires monitoring their activities in three dimensions (3D) with single-cell resolution. Several researchers have proposed various volumetric imaging technologies. However, most technologies require large-scale and complicated optical setups, as well as deep expertise for microscopic technologies, resulting in a high threshold for biologists. In this study, we propose an easy-to-use light-needle creating device for conventional two-photon microscopy systems. By only installing the device in one position for a filter cube that conventional fluorescent microscopes have, single scanning of the excitation laser light beam excited fluorophores throughout over 200 μm thickness specimens simultaneously. Furthermore, the developed microscopy system successfully demonstrated single-scan visualization of the 3D structure of transparent YFP-expressing brain slices. Finally, in acute mouse cortical slices with a thickness of approximately 250 μm, we detected calcium activities with 7.5 Hz temporal resolution in the neuronal population.

## Introduction

Confocal laser scanning microscopy (CLSM) and two-photon excitation laser scanning microscopy (TPLSM) enable the cross-sectional fluorescence imaging of dynamic molecular and cellular phenomena in living specimens^1,2^. Because of its superior penetration depth and less invasiveness owing to its near-infrared (NIR) excitation laser wavelength in comparison to the wavelength of single-photon excitation-based CLSM, the TPLSM is used for thick biological specimens. For visualizations of the 3D microstructures of the specimens, systems generally require *z-*scanning methods, such as a focus drive motor or a piezo-actuator for changing the relative positions between the objective lens and the specimens. Therefore, the temporal resolution for volumetric imaging primarily depends on the mechanical speed of *z*-scanning, which generally requires at least a few seconds^3–5^. However, for 3D visualization of biological processes in living organisms, a considerably higher temporal resolution is usually required. For example, capturing calcium transients and/or tracking the dynamics of subcellular structures (e.g., activities across dendritic spines) on a sub-second resolution scale is critical for in vivo imaging of neuronal activities. Besides fluorescence microscopy, sophisticated phase microscopy such as digital holographic microscopy has been developed for 3D visualization of non-fluorescent biological specimens with high spatiotemporal resolution^6,7^. Such technologies can be concurrently used with fluorescence microscopy, enabling superimpose of fluorescent signals into 3D images. However, since most phase microscopy requires transmitted light via the specimen for 3D image reconstructions, it is difficult to apply the large-scaled, thick specimens such as living animals.

To meet these requirements, cutting-edge fluorescence microscopic technologies for fast volumetric imaging have been proposed^8^. Among them, the Bessel beam-based TPLSM engineers point spread functions (PSFs) to increase the effective depth of field (DOF)^9–12^, which enables the acquisition of projection images by scanning a needle-shaped excitation formed by a Bessel beam at the focus. This technique is emerging as a viable option for imaging larger tissues such as the brain. To achieve single-scan projection imaging with a Bessel beam, the spatial distribution of the excitation light beam was converted into an annular shape before focusing. In principle, an annular-shaped beam must be formed at the pupil plane of an objective lens or at its optically relayed position. However, these ideal positions are typically inaccessible to commercially available TPLSM systems. Thus, realizing light-needle imaging in the TPLSM system requires a thorough understanding of the complicated optical system to be modified, the installation of costly custom-made systems, or the construction of a microscope setup from scratch. Because of these factors, most biologists find it difficult to use light-needle imaging in TPLSM systems.

Here, we propose a method for realizing volumetric imaging with an extended DOF in a TPLSM using a newly developed, easy-to-use, light-needle creating device that can be readily installed in commercially available microscope systems. Simply inserting the device into one of the filter turret positions in an upright microscope results in an axially elongated PSF extended by a factor greater than 70 compared to that of a conventional TPLSM. Furthermore, the developed microscopy system successfully demonstrated single-scan visualizations of calcium activities with 7.5 Hz temporal resolution in an acute jGCaMP7 expressing mouse brain slice with a thickness of ca. 250 μm.

## Results

### Design of the light-needle creating device and effective DOF evaluation

**Figure 1:**
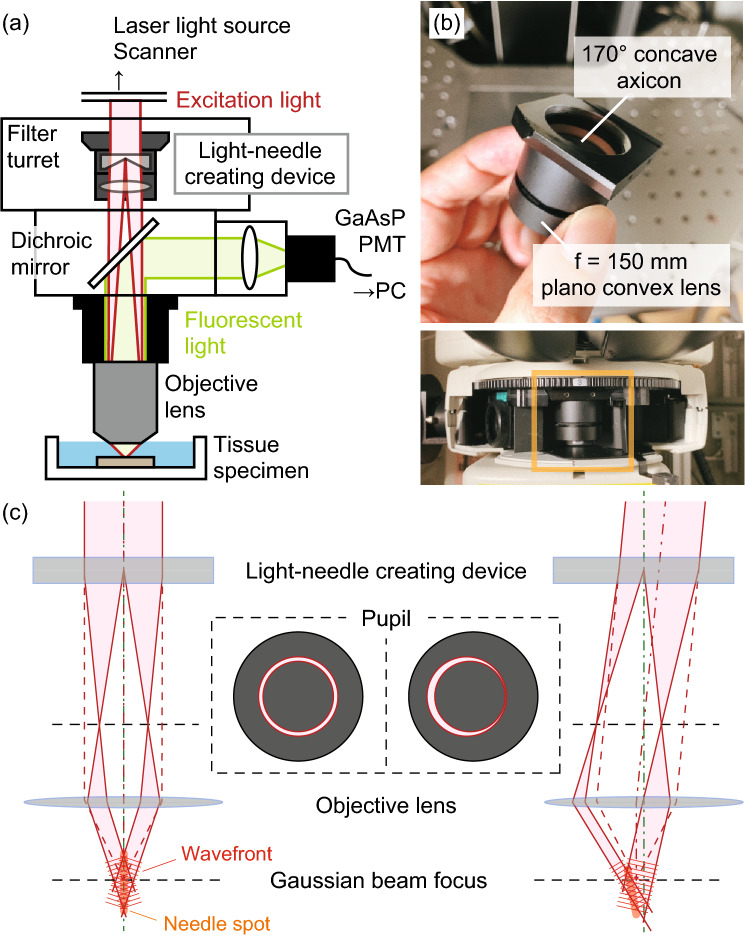
(**a**) Optical schematic of the developed light-needle microscopy. (**b**) Photograph of a light-needle creating device placed in a filter turret of an upright microscope. (**c**) Schematic of the generation of a light-needle spot for the nominal incidence (left) and the oblique incidence (right) to the device, under raster-scanning of an excitation laser light beam.

Figure 1a shows a schematic of the optical setup. A light-needle creating device is a key component for generating a needle-shaped excitation spot at the focus of an objective lens in our TPLSM system. This device, which consists of a concave axicon and a plano-convex lens, is directly inserted into one position of the filter turret of the upright microscope of our TPLSM system (Fig. 1b). The concave axicon converts an incident Gaussian beam into an annularly spreading beam owing to the refraction by the conical surface. The converted beam then forms a thin annular-shaped pattern at the focus of the convex lens built into the stacked device. In our setup, we used a concave axicon with an apex angle of − 170° to generate an annular-shaped beam with an inner diameter of approximately 12 mm at the position of the objective lens. The inner diameter was 68% of the objective lens pupil (17.6 mm). The plano-convex lens had a focal length of 150 mm, which corresponded to the physical distance between the central position of the device and the entrance position of the objective lens. The resultant annular-shaped beam was focused by an objective lens to form a needle-shaped two-photon excitation spot. Fluorescence emission from fluorophores existing in the focal volume was collected by the same objective lens and reflected by a dichroic mirror toward a non-descanned detector before the light-needle creating device.

In previously reported TPLSM systems employing wavefront modulation, the beam-shaping component was placed at an appropriate position corresponding to the pupil of an objective lens using relaying optics^8–12^ or in the vicinity of the pupil^13,14^. Although the use of a Bessel beam in TPLSM is well-established, most of the previous works have employed complicated relaying optics as well as an axicon to generate a Bessel beam^9,10^. In contrast, our device minimally consisting of an axicon and a lens was inserted at a position different from that of the pupil and one slot of the filter turret. This configuration provides a remarkable simplicity of adoption, however for raster scanning, one must consider the position-dependent inclination of a light-needle, as shown in Fig. 1c. When the beam was perpendicular to the center of the device, the needle spot was parallel to the optical axis. On the contrary, scanning an excitation beam caused an oblique incidence to the device, resulting in an annular-shaped beam with a distorted intensity distribution and a lateral offset on the pupil. Consequently, the axis of the needle spot was tilted in the direction opposite to the incident angle.

**Figure 2:**
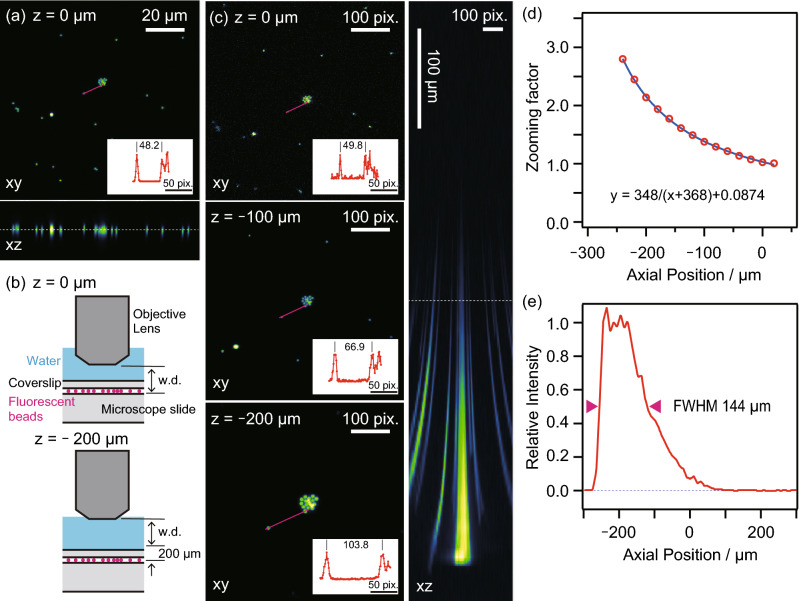
TPLSM images of fluorescently labelled beads on a coverslip. (**a**) *Xy* image at the focus (*z* = 0 µm) and reconstructed *xz* image from *z-*stacks (30 µm thick) measured by the Gaussian beam. (**b**) Relative configurations of the objective lens and the specimen. (**c**) *Xy* image at the focus, 100 and 200 µm position apart from the focus and reconstructed *xz* image from *z-*stacks (600-µm thick) measured by the Bessel beam created by the light-needle creating device. The inset panels indicate the fluorescence intensity profiles along the red lines in the *xy* images. The scale bars along *xy* images were shown as their pixels. (**d**) Zooming effect of the Bessel beam scanned images depending on the position from the focus. (**e**) The axial fluorescence intensity profile of TPLSM image measured by the Bessel beam.

We first measured the TPLSM images of fluorescent beads fixed on a coverslip to evaluate the effective DOF elongation induced by our light-needle creating device. First, a conventional *xyz* image is acquired without the device, as shown in Fig. 2a. The incident Gaussian beam was then converted into the Bessel beam using the light-needle creating device, and the same specimen was measured by changing the relative position to the objective lens (Fig. 2b). As shown in Fig. 2c, the *xy* image at the 0 µm position acquired with the device looked similar to the Gaussian beam excited image in Fig. 2a, except for the marginal region of the field of view (FOV), which could be distorted by the field curvature induced by the device. To evaluate the extension of the DOF, we measured a stacked image of the same FOV for a *z* range of 600 µm. The TPLSM used the light-needle creating device to record the bead image without blurring for the *z* range reaching 200 µm, indicating a remarkable extension of the DOF. However, the TPLSM images were magnified by parting from the focal position because of the tilt of the PSF depending on the angle of the incident beam (Fig. 1c). To evaluate the relationship between the zooming factor and distance from the focal position, the distance between two individual beads was measured as the pixel number for every *z* position. The zooming factor was calculated and plotted against the distance from the focus by comparing every distance with that from the Gaussian beam-scanned image (Fig. 2d). Considering that the tilt angle of the PSF increases linearly with the distance from the center of the FOV, the effective pixel pitch decreases linearly from the ideal pixel pitch as observation depth increases. In other words, the zooming factor, defined as the reciprocal of the size ratio of the effective FOV to the ideal FOV, was inversely proportional to the depth (blue fitting curve in Fig. 2d). The length of the created light-needle was estimated using the axial fluorescence intensity profile shown in Fig. 2e. Because the shape of the axial intensity profile was asymmetric to the position of the maximum intensity, the full-width at half-maximum (FWHM) was determined by the distance between the two half-maximum points, each of which was obtained from the line approximating 7 data points around the half maximum of the fluorescent intensity, which corresponded to the 15 µm range, and confirmed that the FWHM was 144 μm. This value clearly indicates the remarkable extension of the DOF, corresponding to more than 70 times the axial PSF size determined by imaging a 0.2-µm fluorescence bead without the device.

### Fine structure imaging of clearing brain slices with light-needle creating device

**Figure 3:**
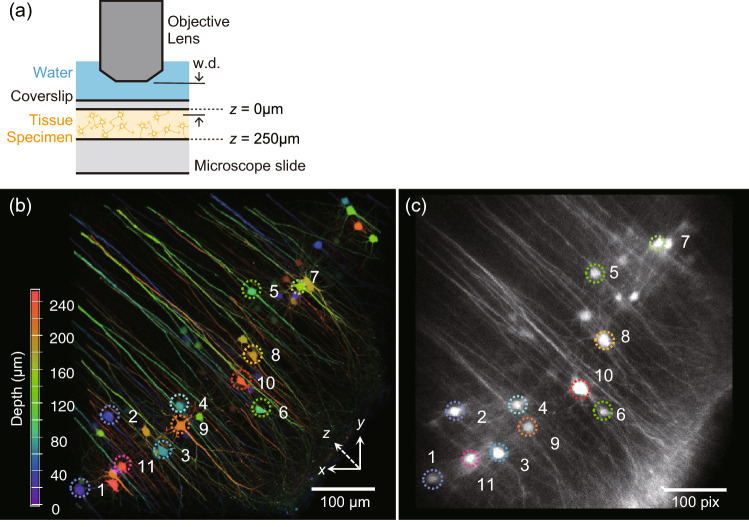
Comparison between *z-*stacked Gaussian beam scanned image and Bessel beam scanned image of a Sca*l*eA2 cleared thy1-YFP-H mouse brain slice. (**a**) Relative configurations of the objective lens and the fixed slice. (**b**) 3D reconstructed image from Maximum intensity projection of a 250 µm thick *z-*stack image of neurons (color-coded by depth) in the sensory cortex measured by Gaussian beam scanning. The neurons were serially numbered in accordance with the *z* position started from the surface of the brain slice. (**c**) A *xy* image of the same field of view in (**b**) at the focus, 60 µm apart from the slice surface, was collected by scanning a Bessel beam using the light-needle creating device. The same neurons were selected and numbered as in (**b**). The scale bar along an image was shown as their pixels.

After characterizing the effective DOF using fluorescence bead measurements, we validated the performance of our volumetric imaging method in biological specimens. A coronal section of the Thy1-YFP-H mouse brain containing the sensory cortex was fixed and treated with Sca*l*eA2 clearing solution. Sca*l*eA2 is an aqueous reagent that clears fixed biological tissue without quenching the fluorescent signals while also reducing light scattering in thick specimens^15^. First, a *xyz* image was captured throughout the 250-μm thickness specimen using conventional Gaussian beam scanning, and the relative configuration of the objective lens and fixed slice is shown in Fig. 3a. Next, a single *xy* image was obtained using Bessel beam scanning generated by our light-needle creating device. The soma and neurites in the Bessel beam image are easily identified, and their depth can be reconstructed by comparing it to the maximum intensity projection image of the *z*-stack Gaussian beam scanning (Fig. 3b,c). As shown in Fig. 3c, the soma located deep in the slice was zoomed (e.g., cell number 10), whereas the soma near the slice surface (e.g., cell number 1) appeared similar to the Gaussian beam-excited image. This observation assumed that a similar depth-dependent zooming effect occurred under Bessel beam scanning, as observed in the bead imaging (Fig. 2). To confirm this phenomenon (Supplementary Fig. S1a), we converted the original Gaussian *z*-stack into an averaged intensity projection image and processed it using a home-built ImageJ plug-in by considering the depth-dependent zooming effect (Fig. 2e). The reconstructed Gaussian image was more correspondence to the Bessel beam *xy* image, except for the edge region of the fixed slice (Supplementary Fig. S1b–e), which might have been distorted by the field curvature induced by the device. Although this additional distortion should be considered for reconstructing more accurate images, such reconstructions will be helpful for assignments of the target in more complicated and/or denser structure. As demonstrated in this experiment, we achieved a volumetric image with fine structures in thick, clearing, fixed brain slices using our light-needle creating device.

### Time-lapse volumetric imaging of neuron dynamics in acute slices

**Figure 4:**
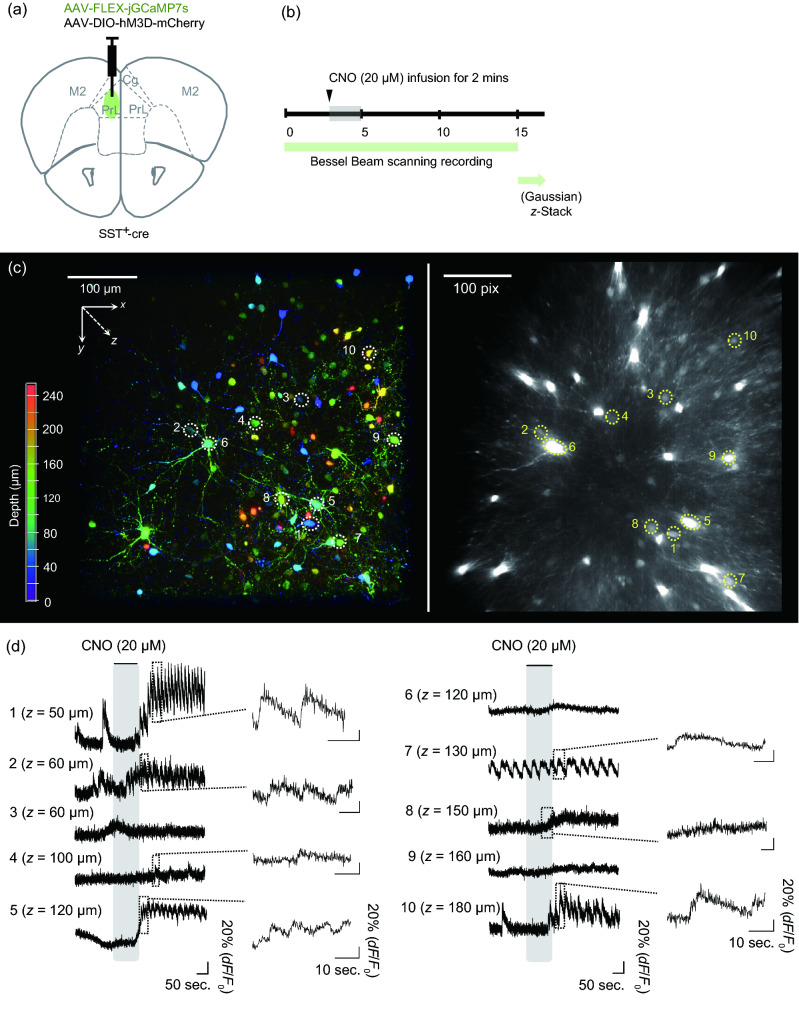
Calcium imaging of acute mouse brain slices. (**a**) Virus injection strategy for expressing calcium probes and chemogenetic tools in SST^+^ interneurons in the prefrontal cortex of SST-cre mice. (**b**) The timeline of calcium imaging and chemogenetic manipulation. (**c**) Comparison between *z-*stacked Gaussian beam scanned image and Bessel beam scanned image. (left) 3D reconstructed image from maximum intensity projection of a 250 µm thick *z-*stack image of SST^+^ neurons (color-coded by depth) measured by Gaussian beam scanning. The neurons were serially numbered in order of the depth from the slice surface. (right) Average intensity projection rendered from time-lapse imaging data. The same field of view in (left) was obtained at 60 µm apart from the slice surface by Bessel beam using the light-needle creating device. The same neurons were numbered as in (left). The scale bar along an image in (left) was shown as their pixels. (**d**) Spontaneous and stimulated calcium traces of individual neurons numbered in (**c**) before, during and after CNO infusion. Magnified traces showed the representative calcium events.

To demonstrate the feasibility of our volumetric imaging method in living organisms, we applied this technique to calcium imaging of acute adult brain slices. For this purpose, somatostatin-expressing (SST^+^) interneurons in the medial prefrontal cortex (mFPC) of SST-cre mice^16^ were labelled with Cre-inducible adeno-associated virus (AAV) encoding the calcium indicator jGCaMP7s. Simultaneously, we expressed hM3Dq, an engineered human muscarinic receptor, in the same region to artificially and selectively activate SST^+^ interneurons using designer receptors exclusively activated by designer drugs (DREADD)^17^ (Fig. 4a). Three weeks after the virus injection, acute brain slices containing the mPFC were placed under a TPLSM microscope. We then performed a *xy* Bessel beam scanning recording by introducing our light-needle creating device. Clozapine-N-oxide (CNO) was introduced into the slices through an infusion system during Bessel beam scanning to chemically activate SST^+^ neurons for two minutes (Fig. 4b). The calcium activity was recorded before, during, and after CNO administration. At the end of the experiments, the same region obtained by Bessel beam scanning was then captured by a 250 μm *z*-stack image of Gaussian beam scanning (Fig. 4c-left). Typical examples of neurons were displayed in Fig. 4d. Similar to the results for fixed brain slices, the reconstructed average intensity projection image of the *z-*stacked Gaussian beam scanned image was similar to that of the Bessel beam scanned image (Supplementary Fig. S1f–i). The jGGaMP7s expressing SST^+^ interneurons distributed at different depths were identified from the Bessel beam excited recording, and their locations were resolved by referring to the Gaussian *z-*stack image (Fig. 4c).

Using Bessel beam scanning recordings, we found that SST^+^ interneurons have a variety of spontaneous calcium activity patterns and responses to chemogenetic manipulation (Fig. 4d). Some neurons showed (1) fast calcium transients (cells 1 and 10), (2) calcium oscillations (cell 7), and (3) sustained baseline changes (cells 6, 8, and 9). In most neurons, mixed types of calcium activity were also observed (e.g., cells 2, 4, and 5). The heterogeneous responses of SST^+^ interneurons upon chemogenetic manipulation may correspond to recent studies in which SST^+^ interneurons were observed to have distinct biological properties^18,19^.

In conclusion, we have successfully demonstrated that Bessel beam scanning using our light-needle creating device enables the monitoring and detection of various activities from a population of neurons located in thick specimens.

## Discussions

In this study, we successfully demonstrated that fast three-dimensional biological phenomena can be captured using single *xy* imaging by mounting our easy-to-use light-needle creating device on a conventional TPLSM microscope system (Fig. 1). In particular, we successfully imaged the spontaneous activities before CNO administration and the evoked activities after CNO stimulation by the Bessel beam scanning using the acute slices from SST^+^-cre mice in which only SST^+^ interneurons expressed jGCaMP7s and DREADD (Fig. 4). SST^+^ interneurons constitute 20–30% of GABAergic interneurons in the mPFC. The somata of SST^+^ interneurons are primarily located in layers V and II/III, with dense axonal arborization in layer I^18,19^. SST^+^ interneurons have diverse subgroups with distinct molecular profiles, anatomical features, and electrophysiological properties^16^. Our system showed the ability to trace the SST^+^ interneurons distributed across different depths with diverse activity properties from a large volume (Fig. 4), which is consistent with previous studies, making it a powerful tool for elucidating how different neuronal subtypes respond to and integrate with each other while receiving input signals from other brain regions. Because obtaining the dynamics of neural activity in a population of neurons with high spatial and temporal resolution is crucial, the volumetric imaging techniques proposed here can overcome the limitations of conventional microscopy for elucidating information processing in the neural circuitry.

However, in terms of ease of use, our proposed methodology has some practical limitations. First, the visualized target was zoomed in the actual image depending on its position relative to the objective lens. Moreover, the acquired image was affected by severe field curvature, resulting in a ring-form shadow appearing at the center of the image when we moved the objective lens toward the specimens. These effects were caused by the position of creating the Bessel beam, which was not the conjugate plane of the pupil of the objective lens in the system. Although the effects of the field curvature remained in the reconstructed image, such information should help assign the targets to be traced. As shown in Fig. 2c, the acquired zooming-factor curve was firmly fitted by a reciprocal linear function. This information enables us to reconstruct a projection image from the *xyz* image acquired using the Gaussian beam, which is nearly identical to our single Bessel beam-scanned image, as shown in Supplementary Fig. S1. On the other hand, the tilted Bessel beam has been applied to stereoscopy, enabling 3D image reconstructions^20^. Similarly, as shown in Figs. 3c and 4c, fluorescent signals from similar-sized somas were projected in our Bessel beam-scanned image as depth-dependent sized appearances. In such cases, the axial position of each soma might be estimated without using the *xyz* image acquired using the Gaussian beam, which most Bessel beam-based TPLSM used^9–11^. In addition, the Bessel beam was converted from an incident Gaussian beam using the light-needle creating device at the filter cube position and was introduced into the pupil of the objective lens with a beam divergence of approximately 4.6°. Such a beam divergence shifts the axial focal position of the PSF from the focus of the Gaussian beam and might result in a decrease in the efficiency of the fluorescent signal detection. To parallelize the beam or decrease the divergence angle, the light-needle creating device requires additional optical elements. Because our device was designed to fit in the position of a filter cube of a conventional microscope, a stacked pair of a concave axicon and a plano convex lens mostly occupied its space. Taking these into account, next generation devices using thinner elements, such as diffractive optical elements^21^, can be designed for brighter and easier-to-use volumetric imaging.

Finally, owing to the simplicity of our light-needle creating device, it can be broadly employed for three-dimensional imaging. As we successfully extended the DOF over 200 μm, it benefits the acquisition of volumetric images in considerable larger specimens, such as measuring the neuron activities in the primate brain. Moreover, the application of our device is not restricted to calcium signal measurements. Any structure sparsely labelled with a biosensor can be imaged, and its biological dynamics can be monitored using this approach, such as capturing the fast changes in membrane potentials with voltage sensors among a large neuronal population with single-cell resolution. Using our device with higher laser power in the future also promises the visualization of tiny structures, such as the neuropils in Fig. 4, which assures us to track the translocation of signalling molecules in multiple dendritic spines during long-term plasticity or animal behavior. On the contrary, the application of Bessel beam scanning imaging normally requires the collection of a large number of optical components and a sophisticated setup to line up all parts in a proper order. Therefore, a larger size of the working space is generally considered when building up the system, which impedes the application within the conventional laboratory. Our device has overcome this limitation, which can be easily carried to space-restricted environments, such as the cleanroom in the hospital. It extends the implantation of Bessel beam-based images to clinical research by facilitating the 3D construction of cancer tissue and tumor metastasis.

Collectively, our light-needle creating device potentially reduced the cost and complexity of the Bessel beam scanning-based volumetric technique for broader applications.

## Materials and methods

### Optical setup

A light-needle creating device was composed of a custom-made concave axicon (physical angle = − 170°; Natsume Optical Corp., Iida, Japan) and a plano convex lens (*f *= 150 mm; LA1433-B, Thorlabs, Inc., Newton, NJ), and was inserted into one position of a filter turret of an upright microscope (FN-1, Nikon, Tokyo, Japan). All fluorescence images were obtained using a TPLSM system (A1R-MP+, Nikon) with a water-immersion objective lens (Nikon, Apo LWD 25×/1.10 NA). A Ti:sapphire laser (MaiTai eHP DeepSee, Spectra-Physics, Santa Clara, CA; tunable wavelength: 690–1040 nm, averaged power: 1.7 W at 920-nm, 1.3 W at 950-nm; pulse width: 100 fs, repetition rate: 80 MHz) was used as the excitation laser light source at wavelengths of 920 nm for GCaMP7 and 950 nm for EYFP. All fluorescence signals under a wavelength of 690 nm were detected by non-descanned detectors equipped with GaAsP PMTs (NDD). The sensitivities of the NDD and laser power were adjusted according to the experiments.

### Animal

All animal studies were carried out in accordance with ARRIVE guidelines and all animal care and experimental procedures were approved by the Institutional Animal Care and Use Committee of the National Institute of Natural Sciences and were performed according to the guidelines of the National Institute for Physiological Sciences (Approval No. 20A017 and 20A122). Mice were housed at 22–24 °C with a standard 12 h light–dark cycle and ad libitum access to water and a standard chow. A mouse line of Thy1-YFP-H, characterized previously^22^, was used to prepare Sca*l*eA2 clearing fixation slices. Four to five months old Sst-IRES-Cre (SOM-cre) mice (Stock No: 013044) were used for acute slice recordings.

### Beads sample preparation

Fluorescently labelled beads (Yellow Green, 0.2-μm diameter/Nile Red, 1 μm diameter; Thermo Fisher Scientific, Waltham, MA) were diluted in water (1:3000, v/v), applied dropwise to glass coverslips, and allowed to dry. The coverslips were mounted using a mounting medium.

### Preparation of ScaleA2 clearing fixation slices

The Sca*l*eA2 clearing slices were prepared as previously reported^15^. Briefly, Thy1-YFP-H mice were transcardially perfused with saline followed by 4% paraformaldehyde (PFA, in 0.1 M PB). The brain was removed and subjected to post fixed in 4% PFA (in 0.1 M PB) at 4 °C overnight; then replaced 4% PFA with 0.1 M PB. 250 μm-thick brain slices contained somatosensory cortex were collected in coronal plane using a vibratome (700smz, Campden Instruments, Leicestershire, UK) and incubated with Sca*l*eA2 solution (4 M urea, 10% (w/v) glycerol, and 0.1% (w/v) Triton X-100) at 37 °C for two days. The slices were mounted on a glass slide (0.17 mm) with Sca*l*eA2 solution and imaged using the TPLSM system.

### Stereotaxic viral injections

A mixture of AAV1-hSyn-FLEX-jGCaMP7s virus (104491, Addgene, Watertown, MA; 1 × 10^13^ vg/mL) and AAV1-hSyn-DIO- hM3Dq-mCherry (44361, Addgene; 7 × 10^12^ vg/mL) was injected unilaterally into the medial prefrontal cortex (mPFC). Homozygous female or male Sst-IRES-Cre (3–4 months old) mice were anesthetized with isoflurane and placed into a stereotactic apparatus (RWD Life Science, San Diego, CA). After the incision site was sterilized with ethanol and iodine, the skull was exposed and a small hole was made over the mPFC. 250 nL of mixed viruses (ratio 1:1) were delivered using 10 µL NanoFil syringes with 33-gauge needle (NF33BV, World Precision Instruments, Sarasota, FL) and a syringe pump (Micro4, World Precision Instruments) at 10 nL/min into mPFC (from bregma, anteroposterior: + 2.4 mm; mediolateral: 0.3 mm; dorsoventral: − 1.6 mm). The needle was slowly removed 10 min after the injection to minimize the backflow of the virus. Mice were returned to their home cage for at least three weeks of recovery before the acute slice image.

### Acute slices preparation

We used 4–5 months old SST-Cre mice, and acute coronal brain slices containing the medial prefrontal cortex were prepared according to the procedures described in a previous study^23^. Briefly, mice were deeply anaesthetized with isoflurane and transcranially perfused with pre-chilled NMDG-HEPES aCSF: 92 mM NMDG, 2.5 mM KCl, 1.25 mM NaH_2_PO_4_, 30 mM NaHCO_3_, 20 mM HEPES, 2 mM thiourea, 5 mM Na-ascorbate, 3 mM Na-pyruvate, 0.5 mM CaCl_2_, 10 mM MgSO_4_·7H_2_O and 25 mM glucose, was oxygenated and adjusted to pH 7.4. After decapitation, the brains were rapidly dissected and immersed in cold oxygenated NMDG-HEPES aCSF. The brains were trimmed and glued to the chilled platform of a vibratome (VT1200, Leica, Nussloch, Germany). Coronal slices (250 μm) containing the mPFC were cut in ice-cold NMDG-HEPES aCSF and were continuously bubbled with 95% O_2_ and 5% CO_2_. The slices were transferred to a holding chamber with pre-warm (34 °C) oxygenated NMDG-HEPES aCSF, and NMDG protective recovery procedure was carried out immediately. During this process, the indicated volumes of Na^+^ spike-in solution (2 M NaCl) were added at the indicated times, as described previously^24^, to gradually reintroduce Na^+^ into the brain slice chamber. 30 min later, slices were transferred to a holding chamber containing oxygenated HEPES containing aCSF at room temperature: 92 mM NaCl, 2.5 mM KCl, 1.25 mM NaH_2_PO_4_, 20 mM HEPES, 20 mM NaHCO_3_, 2 mM thiourea, 5 mM Na-ascorbate, 3 mM Na-pyruvate, 2 mM CaCl_2_, 2 mM MgSO_4_·7H_2_O and 25 mM glucose, was titrated and adjusted to pH 7.4. Slices were allowed to recover for at least an hour in the holding chamber before initiating slice recording. Recordings were performed at room temperature (22−23 °C) with constant superfusion (3 mL/min) of oxygenated recording aCSF: 105 mM NaCl, 2.5 mM KCl, 1.25 mM NaH_2_PO_4_, 24 mM NaHCO_3_, 2 mM CaCl_2_·4H_2_O, 2 mM MgSO_4_·7H_2_O, 12.5 mM glucose, and adjusted pH to 7.4.

### Image acquisition and data analysis

The images of the fluorescent beads measured by the Gaussian beam had a pixel size of 199 nm (no binning), FOV size of 102 × 102 μm (512 × 512 pixels), and exposure time of 1 s. The *z-*stack (30 μm-thick) was captured at 0.5 μm intervals. For measurements of the same specimen with the Bessel beam, pixel numbers, scanning range of scanning mirrors, and exposure time were kept with Gaussian, while the *z-*stack (600 μm-thick) was captured at 2.5 μm and 10 μm intervals for evaluating the length of the created light-needle and the zooming effect, respectively.

The images of Sca*l*eA2 clearing slices were constructed by averaging two acquired images for Gaussian beam scanning, having a pixel size of 0.99 μm, an FOV size of approximately 500 × 500 μm (512 × 512 pixels), and exposure time of 1 s. The *z-*stack (250 μm-thick) was captured at 2 µm intervals. For measurements of the same specimen with the Bessel beam, pixel numbers, scanning range of scanning mirrors, and exposure time were kept with Gaussian, while the averaging number was 4.

To measure Ca^2+^ activity in acute slices, fluorescence signals were detected by the TPLSM system with the same pixel numbers and scanning range of scanning mirrors with measurements of Sca*l*eA2 clearing slices, while the exposure time and averaging number were 33 ms and 4, respectively. Spontaneous calcium activity of SST^+^ interneurons was first checked by Gaussian beam scanning to validate the health conditions of the recording slices. The same brain regions were used for both the Gaussian and Bessel beam scanning images. All image data were processed and analyzed using image software (ImageJ and NIS element Ver.4.51, Nikon). The calcium activity of SST^+^ interneurons was extracted from the time-lapse data and analyzed as *dF/F*_0_. In Fig. 4, ten regions of interest (ROIs) corresponding to the soma of SST^+^ interneurons were selected manually. *F*_0_ was calculated as the mean pixel brightness before CNO stimulation; intensities exceeding two standard divisions were excluded while calculating the mean. The related fluorescence change, *dF/F*_0_, was evaluated using the signal intensity ratio between each time point (*F*) and *F*_0_.

To anticipate the Bessel beam scanned image using the light-needle creating device, an image reconstruction macro was written using ImageJ. The macro-converted *z-*stack image was measured using Gaussian beam scanning of the reconstructed image, reflecting the zooming factor depending on the distance between the objective lens and the target (Fig. 2d).

## Supplementary Information

Supplementary Figure S1.

## Data Availability

The datasets generated and/or analyzed during the current study are available from the corresponding authors upon reasonable request.
