# Supplementary material for: Single-scan volumetric imaging throughout thick tissue specimens by one-touch installable light-needle creating device
Source: Sci Rep. 2022 Jun 21;12:10468. doi: 10.1038/s41598-022-14647-3 (PMC9213396; doi:10.1038/s41598-022-14647-3)
Supplement: Supplementary file 1 — Supplementary Figure S1. [file 41598_2022_14647_MOESM1_ESM.pdf]

Supplementary materials for

Single-scan volumetric imaging throughout thick tissue specimens by one-touch installable light-needle creating device

Ching-Pu CHANG, Kohei OTOMO *, Yuichi KOZAWA, Hirokazu ISHII, Miwako YAMASAKI, Masahiko WATANABE, Shunichi SATO, Ryosuke ENOKI, and Tomomi NEMOTO*

*** Corresponding authors:**

Kohei Otomo, k.otomo.hd@juntendo.ac.jp,

Tomomi Nemoto, tn@nips.ac.jp

Supplementary Figure

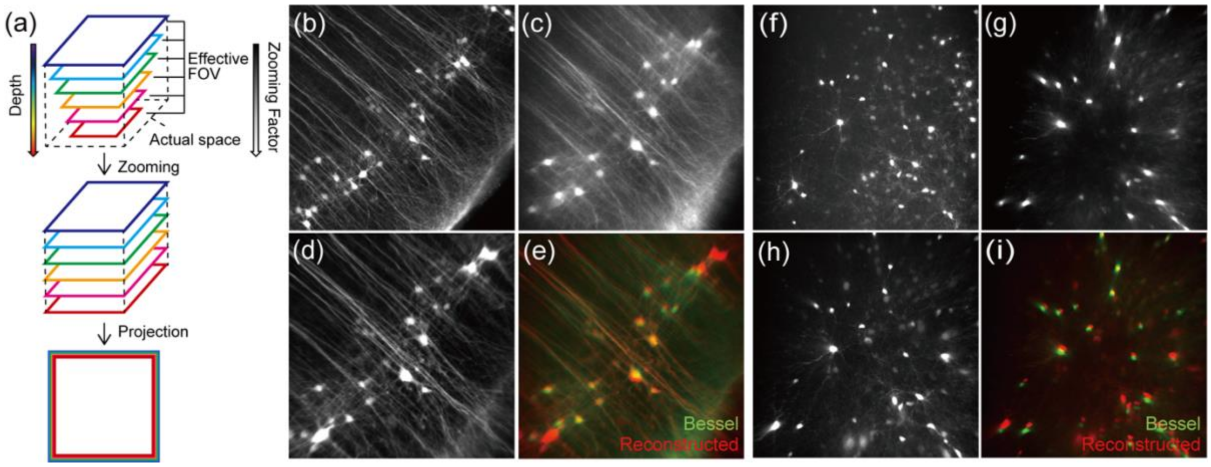

Fig. S1. Comparison between *z*-stacked Gaussian beam scanned image, Bessel beam scanned image and reconstructed Gaussian beam scanned image based on the function about the zooming factor. (a) A schematic illustration of an acquired Bessel-beam scanned image by using the light-needle creating device. (b) Average intensity projection image of *z*-stack Gaussian from Fig. 3b. (c) A *xy* image of Bessel beam image from Fig. 3c. (d) Reconstructed average intensity projection of z-stacked Gaussian from Fig. 3b. (e) Merged image of reconstructed Gaussian image (Red) and Bessel beam image (green). (f) Average intensity

projection image of *z*-stack Gaussian from Fig. 4c-left. (g) Average intensity projection rendered from time-lapse Bessel beam imaging data from Fig. 4-right. (h) Reconstructed average intensity projection of *z*-stacked Gaussian from Fig. 4c-right. (i) Merged image of reconstructed Gaussian image (Red) and Bessel beam image (green).
